# Prediction of outcome in locally advanced breast cancer by post-chemotherapy nodal status and baseline serum tumour markers

**DOI:** 10.1038/sj.bjc.6600616

**Published:** 2002-11-26

**Authors:** B Brenner, N Siris, E Rakowsky, E Fenig, A Sulkes, H Lurie

**Affiliations:** Institute of Oncology, Rabin Medical Center, Beilinson Campus, Petah Tiqva, Israel; Sackler Faculty of Medicine, Tel Aviv University, Tel Aviv, Israel

**Keywords:** locally advanced breast cancer, tumour markers, prognostic factors

## Abstract

In spite of the apparent improvement in outcome in locally advanced breast cancer, the prognosis remains dismal in many patients. The aim of this study was to define prognostic subgroups within this heterogeneous entity. Between 1990 and 1999, 104 consecutive patients with locally advanced breast cancer were treated by a multimodality programme consisting of 4–6 courses of CAF induction chemotherapy followed by surgery, breast-conserving when feasible. In most cases, chemotherapy was then resumed, up to a total of eight courses, followed by locoregional radiation therapy. Patients with hormone receptor-positive tumours received tamoxifen (20 mg day^−1^) for 5 years. At a median follow-up of 57 months, the 5-year overall survival for the entire group and the disease-free survival for the 94 operated patients were 65% and 53%, respectively. Univariate analysis identified 10 prognostic factors of overall and disease-free survival, of which four retained significance on multivariate analysis: inflammatory breast cancer (*P*=0.0000, *P*=0.0004, respectively), baseline tumour markers (*P*=0.003 for both), post-chemotherapy number of involved nodes (*P*=0.003; *P*=0.017) and extracapsular spread (*P*=0.052; *P*=0.014). In conclusion, besides inflammatory features, baseline tumour markers and post-chemotherapy nodal status are strong predictors of outcome in locally advanced breast cancer.

*British Journal of Cancer* (2002) **87**, 1404–1410. doi:10.1038/sj.bjc.6600616 www.bjcancer.com

© 2002 Cancer Research UK

## 

Locally advanced breast cancer (LABC) accounts for 10–29% of all breast carcinomas in the Western World (Lippman *et al*, 1986; Valero *et al*, 1996). With the introduction of multimodality therapeutic strategies, 5-year disease-free survival rates in the order of 30–70% have been reported, with overall survival ranging between 35 and 80% (Hortobagyi and Buzdar, 1991; Hortobagyi *et al*, 1996). Overall, distant failure and early death are not uncommon, emphasizing the need for more effective treatments.

LABC is a heterogeneous group of tumours, ranging from neglected slow-growing neoplasms to rapidly proliferating and aggressive ones. Therefore, a uniform treatment approach is doomed to fail. To better define patient subgroups and to select the more appropriate treatment options, intensive efforts have been invested to identify prognostic factors. Researchers agree that the presence of inflammatory breast cancer (IBC) (Jaiyesimi *et al*, 1992; Palangie *et al*, 1994) and poor pathological response to neoadjuvant chemotherapy (Feldman *et al*, 1986; Sataloff *et al*, 1995; Honkoop *et al*, 1998) yield a worse outcome. Conclusions regarding other possible factors are conflicting.

The present retrospective prognostic factor analysis included a relatively homogeneous group of 104 patients with LABC treated in one institution by a uniform policy during a fairly short period of time.

## MATERIALS AND METHODS

### Patients

The study sample consisted of 104 consecutive patients with LABC who were treated at Rabin Medical Center from 1990 to 1999. LABC was defined as histologically or cytologically documented American Joint Committee on Cancer (AJCC) (Fleming *et al*, 1997) stages IIB, IIIA, IIIB or IV (with ipsilateral supraclavicular lymph node involvement only) breast cancer. Before the onset of treatment, all patients underwent a baseline work-up, as follows: complete history and physical examination, complete blood count, blood chemistry analysis, serum CEA and CA-15.3 levels, chest X-ray, abdominal computerized tomography or ultrasonography, bone scan, bilateral mammography, and cardiac scintigraphy (MUGA).

### Treatment programme

On confirmation of the diagnosis, patients were offered a multimodality treatment programme consisting of 4–6 courses of induction chemotherapy followed, when maximal response was achieved, by surgery. Thereafter, adjuvant chemotherapy was administered to an overall total of eight courses followed by locoregional radiation therapy. Patients with hormone receptor-positive tumours also received adjuvant tamoxifen (20 mg day^−1^) for 5 years.

### Chemotherapy

Preoperative chemotherapy consisted of cyclophosphamide 600 mg m^−2^, doxorubicin 60 mg m^−2^, and 5-fluorouracil 600 mg m^−2^ (CAF protocol), all administered as a rapid intravenous infusion on day 1 of each cycle and repeated every 21 days for up to eight courses, until a maximal clinical response was achieved. The clinical response was categorized on the basis of the physical examination: complete – total resolution of the breast mass and axillary adenopathy; partial – at least a 50% reduction of the product of the two largest perpendicular dimensions of the breast mass and axillary adenopathy: minor – 25% – 50% reduction of the product of the two largest perpendicular dimensions of the breast mass and axillary adenopathy; stable disease – less than 25% change in the product of the two largest perpendicular dimensions of the breast mass and axillary adenopathy; progressive disease – more than 25% increase in tumour size. The pathological response was defined as complete when there was no microscopic evidence of residual tumour, or as microscopic (intraductal or invasive) or macroscopic.

After surgery, the CAF protocol was resumed to a total of eight courses, unless there was disease progression during treatment. In three patients, other regimens were used postoperatively: experimental high-dose treatment followed by autologous stem cell support, or four courses of paclitaxel every 3 weeks, or methotrexate instead of doxorubicin because of impaired cardiac function. Patients underwent a physical examination after each course, and systemic work-up, including cardiac scintigraphy, at the end of both preoperative and postoperative chemotherapy.

### Surgery

The standard surgical procedure was a modified radical mastectomy. However, when feasible, breast-conserving surgery, either lumpectomy or quadrantectomy, with axillary lymph node dissection was performed. The ultimate decision on the type of surgery was left to the discretion of the treating physician and the patient. Surgical specimens were analysed for tumour histology, nodal status, grade, and hormone receptor status.

### Radiotherapy

Radiotherapy was used as adjuvant treatment following surgery in 94 patients, or for definitive treatment of the residual mass in four patients. Six patients did not receive radiation treatment owing to the rapid progression of metastatic disease which necessitated immediate systemic treatment. Radiation therapy to the chest wall or residual breast tissue was administered by two tangential fields using a megavoltage photon beam (6 MV). When breast-conserving surgery was performed, the tumour bed received a radiation boost with an electron beam (dose specified to the 90% isodose line). For supraclavicular and axillary lymph nodes, a direct field was used. The planned total dose to the tumour bed was 60 Gy, and to all other fields, 50 Gy. Treatment was delivered with a 6 MV linear accelerator in daily fractions of 2 Gy each, 5 days a week.

### Follow-up

After completing the primary treatment plan, patients were followed every 3 months for the first 2 years, every 6 months in the next 3 years, and annually thereafter. Follow-up included physical examination, blood chemistry and evaluation of tumour markers. Chest X-ray, upper abdominal ultrasonography and mammography were done yearly. If recurrent disease was suspected, a complete staging work-up was performed.

### Statistical analysis

Overall survival (OS) was defined as the interval between the date of diagnosis and the date of death/last date known to be alive. Disease-free survival (DFS) was defined according to the Manual of the European Organization of Research and Treatment of Cancer (Eortc Breast Cancer Cooperative Group, 2000) as the interval between the date of surgery, or in patients with pathological complete response, the date on which complete clinical response was achieved, and the date of first evidence of recurrence. The analysis of OS included all 104 patients, and of DFS, only those 94 patients who underwent surgery and were thus rendered free of disease. Both rates were estimated by the Kaplan-Meier product limit method (Kaplan and Meier, 1958). Three patients who died of causes other than breast cancer (second malignancy, myocardial infarction, or bronchial asthma) were considered lost to follow-up. Comparisons between subgroups according to patient, tumour and treatment variables were performed with the log-rank test. Cox proportional hazard regression models (Cox, 1972) were applied for multivariate analysis. Risk ratios and 95% confidence intervals (CI) were calculated from the model. SPSS software was used to perform these tests.

## RESULTS

### Patient and tumour characteristics

The clinical characteristics of the patient population at presentation are depicted in Table 1

**Table 1:**
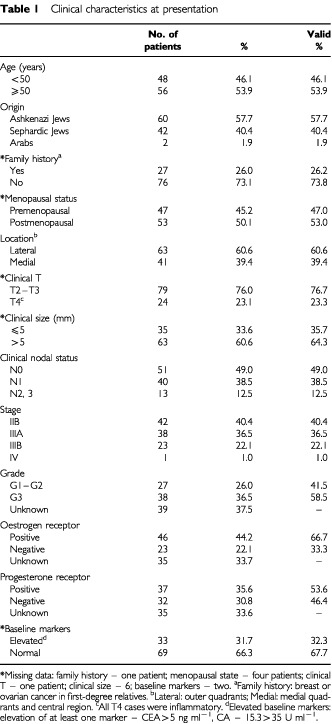
Clinical characteristics at presentation

. Median age was 50 years (range 29–72 years). Sixty patients (57%) were of Ashkenazi origin, and 49 (48%) had a history of malignancy in first degree relatives, the most common of which was breast cancer (24 patients). Sixty-four per cent of the tumours were clinically staged as T3, and 23% had pathological (cutaneous lymphatic tumour emboli) or clinical (red-brownish diffuse discolouration and oedema of the skin) features consistent with inflammatory carcinoma. About half the patients presented with axillary lymphadenopathy. Two-thirds of the tumours were evaluated for grade and hormone receptor expression. Thirty-six per cent were found to be poorly differentiated, and 44% of them were hormone-receptor-positive. Thirty-three patients (32%) had an elevated baseline serum CEA (>5 ng ml^−1^) (median 10.9; range 5.4–128.3 ng ml^−1^) and/or CA-15.3 (>35 U ml^−1^) (median 48; range, 35.1–268.8 U ml^−1^) level.

### Treatment

Patients received 3–8 courses of neoadjuvant CAF (median, six courses); 101 patients (97%) received at least four courses. Three patients did not complete the induction phase because of disease progression (one patient) or refusal (two patients). The objective clinical response rate of the entire group was 76%, with 31% achieving complete clinical response. Accordingly, 94 of the 104 patients (90.4%) underwent surgery. In 40 patients (38%), the procedure was breast-conserving. Ten patients (9.6%) did not undergo surgery because of suspected or overt systemic disease (seven patients), refusal (two patients), or residual unresectable disease (one patient).

Pathological examination of the primary tumour revealed a complete pathological response in 12 patients (11.5%); 10 patients had microscopic invasive disease and one, *in situ* residual disease. Out of the 88 patients who underwent axillary lymph node dissection (median number of nodes examined – 14, range 2–34), 56 (64%) were found to have metastatic nodal involvement (median number of involved nodes – 2, range 1–21). None of the patients who achieved a complete pathological response in the breast had residual axillary lymph node involvement.

### Overall and disease-free survival

At a median follow-up of 57 months (range 19–123 months), 55 patients (53%) were alive with no evidence of disease and nine patients (9%) were alive with disease. Three patients (3%) died without any evidence of breast cancer. The cause of death in each of these patients was second malignancy, bronchial asthma and myocardial infarction. Thirty-seven patients (35%) died of disease. The 5- and 10-year OS rates for the entire group of 104 patients were 65% and 46%, respectively. For patients who achieved complete pathological response the 5-year OS reached 92%. The median OS (104 patients) was 88 months.

Out of the 47 patients (45%) with recurrent disease, 46 had systemic involvement, of whom 12 (11%) had both locoregional and distant metastases. The 5-year DFS rate for the 94 patients who underwent surgery was 53%. Median DFS has not been reached.

### Prognostic factors

#### Univariate analysis

The univariate analysis of the pretreatment clinical prognostic factors is shown in Table 2

**Table 2:**
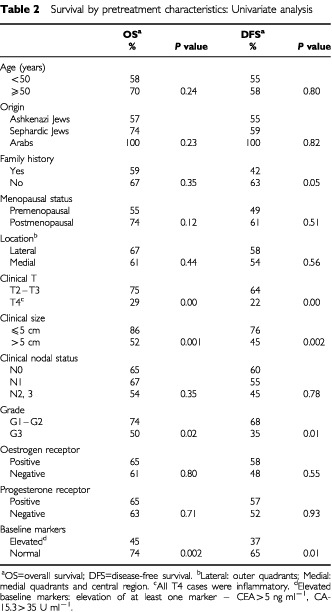
Survival by pretreatment characteristics: Univariate analysis

**Figure 1:**
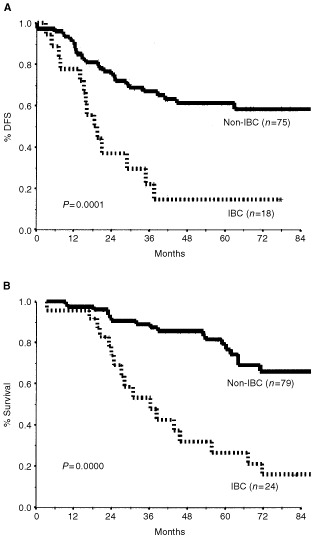
Disease-free survival (**A**) and overall survival (**B**) by presence of inflammatory breast cancer (IBC).

**Figure 2:**
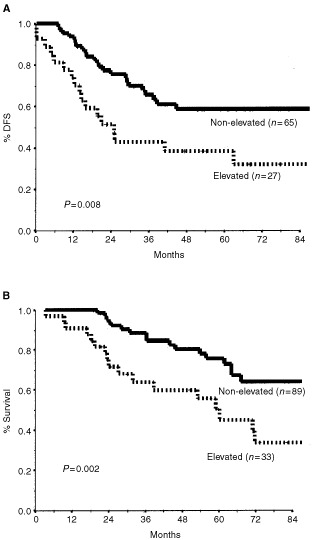
Disease-free survival (**A**) and overall survival (**B**) by baseline tumour markers.

. Four factors were found to significantly correlate with poorer outcome (OS and DFS): clinical tumour size larger than 5 cm, inflammatory features, high grade, and elevated levels of baseline markers. Inflammatory carcinoma had the strongest impact, decreasing the estimated 5-year OS from 79 to 27% (Figure 1

). Elevated tumour markers were associated with a decrease in 5-year OS from 76% to 45% (Figure 2

).

The univariate analysis of the treatment-related prognostic factors are shown in Table 3

**Table 3:**
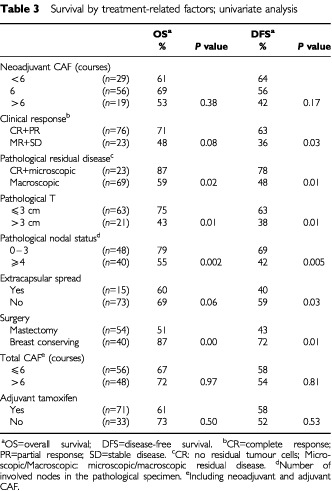
Survival by treatment-related factors; univariate analysis

**Figure 3:**
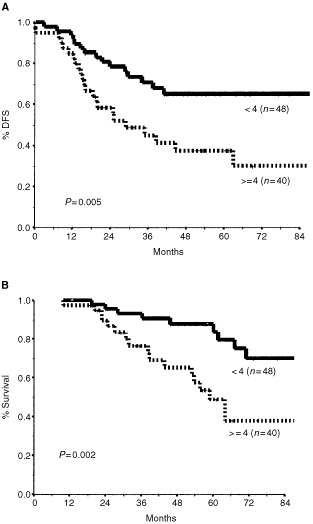
Disease-free survival (**A**) and overall survival (**B**) by number of metastatic axillary lymph nodes.

**Figure 4:**
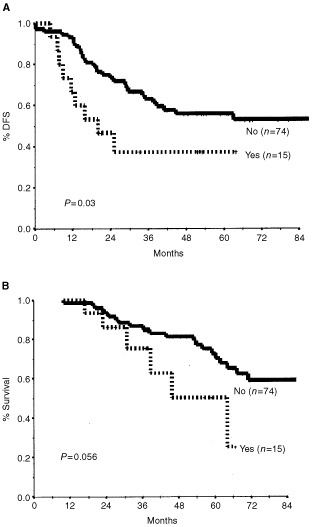
Disease-free survival (**A**) and overall survival (**B**) by extracapsular nodal spread.

. Clinical response was found to significantly predict only DFS, and its practical consequence, that is, the decision to perform breast-conserving surgery, seemed to be significantly correlated with both OS and DFS. Four pathological measures of response correlated with a favourable outcome (OS and DFS): complete pathological response, residual tumour of 3 cm or smaller, fewer than four positive residual lymph nodes, and absence of extracapsular nodal spread. Meticulous analysis of the number of residual lymph nodes (data not shown) indicated the cut-off of four to be the most predictive of outcome: the presence of four or more metastatic axillary lymph nodes was associated with a decrease in 5-year OS from 84 to 49%, and in DFS from 65 to 38% (Figure 3

). Extracapsular spread was also predictive for both of these measures, as illustrated in Figure 4

.

### Multivariate analysis

Of the various multivariate models of OS and DFS, the one depicted in Tables 4

**Table 4:**
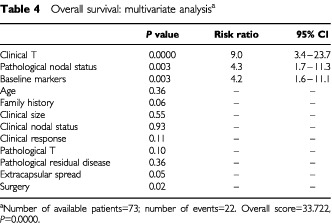
Overall survival: multivariate analysis^a^

and 5

**Table 5:**
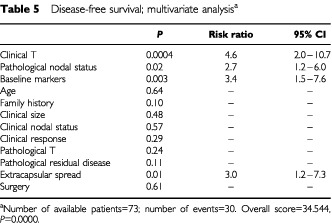
Disease-free survival; multivariate analysis^a^

was chosen because of its clinical relevance, high statistical power, and high availability of patients and events. According to this model, four factors retained statistical significance: two of the clinical factors available at presentation, namely, inflammatory characteristics and baseline tumour markers, and two of the treatment-associated factors, namely, number of involved nodes and extracapsular spread. The latter had only borderline significance in the OS model.

## DISCUSSION

The clinical characteristics of our patient population with LABC largely resemble those of previous reports (Fein *et al*, 1994; Karlsson *et al*, 1998), except for the relatively high rate of hormone receptor-positive tumours (67%). However, this last figure should be viewed with caution, as hormone receptors were examined in only two-thirds of the cases, either because the tumours were unoperated or complete pathological response was achieved. The clinical and pathological response rates in our patients to neoadjuvant CAF chemotherapy are in accordance with other reported series (Delena *et al*, 1978; Lippman *et al*, 1986). The high overall clinical objective response rate of 76% allowed for the fact that 43% of the surgical procedures were breast-conserving. The 5- and 10-year OS for the entire group (65% and 46%), as well as the median OS (88 months), are within the upper range of other published studies (Hortobagyi and Buzdar, 1991; Weshler *et al*, 1990). Whether these figures are incidental or reflect biological or treatment-related phenomena, such as a relatively high percentage of hormonal receptor-expressing tumours or a rather strict treatment frame, is unclear.

Univariate analysis identified several variables, some pretreatment and some treatment-related, as prognostic factors for OS and DFS. On multivariate analysis, only four retained statistical significance: inflammatory features, baseline tumour markers, number of involved nodes, and extracapsular spread of malignant cells.

Inflammatory breast cancer is known to have an aggressive clinical course, very often resulting in early recurrence and death (Pierce *et al*, 1992; Sanchez-Forgach *et al*, 1992). Some authors have argued against the inclusion of patients with IBC in studies of LABC because of their clearly worse outcome (Karlsson *et al*, 1998). In our study, too, inflammatory changes were associated with a significant decrease in 5-year survival rates from 79 to 27% (*P*=0.000) (Figure 1). This emphasizes the inadequacy of current treatment methods to meet the challenge posed by this entity. It may also justify separating IBC from other types of LABC.

Serum levels of tumour markers correlate with the stage of the disease in breast cancer (Fujino *et al*, 1986; Safi *et al*, 1991). However, owing to the low sensitivity and specificity of these tests, they are usually regarded as useful only for follow-up after primary therapy, and for monitoring response to treatment of metastatic disease (van der Linden *et al*, 1985; Cheung *et al*, 2000). However, in the present study, we found that baseline serum CEA and CA-15.3 levels could predict patient outcome. As illustrated in Figure 2, patients who presented with an elevation of at least one of these markers at diagnosis had a 5-year survival rate of 45%, whereas the rate for patients with markers within normal range was 76% (*P*=0.002). Indeed, elevated levels of tumour markers were associated with a greater than four-fold risk of dying of breast cancer and a greater than three-fold risk of recurrence. To the best of our knowledge, this is the first report to identify the prognostic value of standard tumour markers in LABC. Once validated, tests for baseline tumour markers which are objective, comparable and easily obtainable, may be incorporated into the primary care algorithm of LABC.

We found that the pathological response, particularly the axillary status, is the main determinant of both OS and DFS. Moreover, on multiple Cox regression models, two parameters of axillary involvement were indicative of outcome: the presence of more than three involved lymph nodes and extracapsular spread. While the prognostic importance of the former has been reported before (Kuerer *et al*, 1998; Victor *et al*, 1999), the impact of the latter in this context has not yet been fully recognized.

To identify patients with LABC who are at higher risk of primary treatment failure, we and others (Sanchez-Forgach *et al*, 1992; Honkoop *et al*, 1998) chose OS and DFS as outcome measures. Using this approach, we distinguished two pretreatment and two treatment-related prognostic factors. Other investigators chose other measures of outcome, such as clinical and pathological response to neoadjuvant treatment (Robertson *et al*, 1994; Walker *et al*, 1999) or local control (Pierce *et al*, 1992; Victor *et al*, 1999). A thorough search of the literature revealed only a few studies of LABC in which a formal prognostic factor analysis was performed. Moreover, despite the diversity of outcome measures used, we recognized only a few variables whose impact, regardless of the manner of testing, was significant. In a retrospective analysis of 100 patients, Victor *et al* (1999) correlated the presence of IBC and four or more involved axillary nodes with poorer outcome. Their conclusions were supported by Gardin *et al* (1995) in an analysis of 125 patients, and by others as well (Sanchez-Forgach *et al*, 1992; Kuerer *et al*, 1998; Zambetti *et al*, 1999). The clinical size of the primary tumour (Valagussa *et al*, 1990; Fein *et al*, 1994) as well as the clinical nodal status (Valagussa *et al*, 1990) were also found to correlate with outcome. Two studies identified the prognostic importance of neoadjuvant treatment itself. Karlsson *et al* (1998) found that worse OS and DFS were associated with the receipt of less than 75 and 60%, respectively, of the intended dose intensity. In a phase II study, Honkoop *et al* (1998) noted a detrimental effect of a lower number of preoperative chemotherapy cycles. Response to preoperative treatment in LABC is a rather complex issue. In a large prospective study, Kuerer *et al* (1998) found that achievement of a complete clinical response was associated with better DFS. However, most authors agree that the pathological response is more predictive of survival than the clinical response (Sataloff *et al*, 1995; Feldman *et al*, 1998; Honkoop *et al*, 1986). Many aspects remain unclear in this regard. Is it solely the achievement of a complete pathological response that distinguishes good from bad prognosis, or does the favourable group also include patients with residual microscopic disease? Is it the resolution of the primary tumour that is most important or the extent of the remaining axillary involvement, or both? Of the various histopathological markers tested, only hormone-receptor positivity (Robertson *et al*, 1994), co-expression of Pgp/p53 and marked staining for Ki-67 (Honkoop *et al*, 1998) were found to influence survival.

In conclusion, the present study identifies four prognostic factors of LABC: inflammatory features, number of involved nodes, baseline tumour markers and nodal extracapsular spread. While the prognostic importance of the first two has already been established, the influence of the latter two on overall and disease-free survival of patients with LABC has not yet been fully acknowledged. As a finding of extracapsular spread can influence the decision regarding postoperative adjuvant treatment, the presence of elevated baseline tumour markers may have a substantial impact on the preoperative management too, provided these findings are supported by other trials. In light of the generally disappointing results of current therapies, patients manifesting any of these features should be encouraged to participate in clinical trials.
